# Clinical Outcomes Associated with Stellate Ganglion Block Across Multiple Pain Phenotypes

**DOI:** 10.3390/jcm14217611

**Published:** 2025-10-27

**Authors:** Zeki Boga, Cagatay Kucukbingoz, Ahmet Yilmaz, Semih Kivanc Olguner, Ali Arslan, Mehmet Ozer, Mustafa Emre Sarac, Yurdal Gezercan

**Affiliations:** 1 Department of Neurosurgery, Adana City Training and Research Hospital, Adana 01230, Turkey; kivanc3olguner@hotmail.com (S.K.O.); aliarslan26062006@hotmail.com (A.A.); mehmetozerdr@gmail.com (M.O.); emre_sarac@hotmail.com (M.E.S.); gezercan@hotmail.com (Y.G.); 2 Department of Algology, Adana City Training and Research Hospital, Adana 01230, Turkey; ckbingoz.md@gmail.com (C.K.); dr.ahmetyilmaz27@gmail.com (A.Y.)

**Keywords:** stellate ganglion block, pain management, complex regional pain syndromes, migraine disorders, neuralgia, postherpetic, pain, quality of life

## Abstract

**Background/Objectives**: Stellate ganglion block (SGB) is an interventional technique frequently applied to manage pain associated with sympathetic dysfunction. This study aimed to evaluate the short-term clinical outcomes and tolerability of SGB in patients with different pain phenotypes. **Methods**: From 1 January 2024 through 1 March 2025, 96 patients who underwent fluoroscopy-guided SGB at a single center were retrospectively analyzed. The Oswestry Disability Index (ODI) was used to assess functional status, the SF-36 was applied to evaluate health-related quality of life, and the Visual Analog Scale (VAS) was employed to measure pain intensity. This study included baseline measurements and follow-up evaluations at 1, 3, and 6 months after the procedure. **Results**: Statistically significant improvements were observed in VAS, ODI, and SF-36 scores across all pain groups (*p* < 0.001). The largest median VAS reductions were observed in the migraine (4.0 [3.5–5.0]) and complex regional pain syndrome (CRPS) (3.7 [3.0–4.5]) groups, both exceeding the minimal clinically important difference (MCID). Patients with neuropathic and nociceptive pain showed smaller median reductions (3.4 [2.8–4.0] and 3.0 [2.5–3.8], respectively). The highest proportion of responders (≥50% VAS reduction) was noted in the migraine group (64.3%), while the lowest occurred in the nociceptive group (37.5%). Multivariate analysis identified pain phenotype as the only independent predictor of favorable outcomes, particularly in migraine and CRPS. Minor transient complications occurred in 9.4% of patients, all resolving spontaneously. **Conclusions**: SGB was well tolerated and associated with significant pain reduction and functional improvement. The observed clinical benefits may reflect mechanisms involving both peripheral and central sympathetic modulation. Larger multicenter prospective studies with extended follow-up are warranted to confirm these findings.

## 1. Introduction

A stellate ganglion block (SGB) is an interventional technique performed on the sympathetic system with the aim of temporarily interrupting the sympathetic chain. It has long been used for managing pain in the head, neck, thoracic region, and upper extremities originating from sympathetic sources [1,2]. The procedure is usually performed with ultrasound or fluoroscopy, and a combination of local anesthetics or corticosteroids is used. By blocking the sympathetic system, SGB is associated with vasodilation, increases regional perfusion, and reduces pain intensity [3,4]. SGB is typically performed to manage pain related to postherpetic neuralgia, migraine, vascular headaches, traumatic neuropathic pain, and complex regional pain syndrome (CRPS). However, it has also been used for managing post-traumatic stress disorder and other conditions associated with sympathetic overactivity, such as anxiety disorders, menopausal vasomotor symptoms, and post-COVID-19 syndromes [5,6].

Studies today include few participants and use retrospective methods to examine individual clinical symptoms of pain. Although evaluations of SGB outcomes have been conducted for different types of pain, studies comparing multiple phenotypes within the same cohort remain scarce. The potential clinical relevance of ultrasound-guided SGB for postoperative recovery and functional outcomes has gained increasing attention according to a recent meta-analysis [7]. Research into noninvasive stellate ganglion blockade methods using physical agent modalities also indicates potential utility for managing conditions linked to sympathetic hyperactivity, as shown in a systematic review [8]. These research gaps make it difficult to identify which patient groups show more favorable response patterns to SGB. The clinical response depends on multiple factors, including pain type, illness duration, anatomical location, comorbid conditions, and treatment methods. Clinical outcomes may differ depending on medication selection, imaging techniques, injection frequency, and the occurrence of complications [9,10,11,12]. Therefore, evaluating both technical and clinical variables within a single study population may provide evidence to guide patient selection and optimize practice.

The present study aims to evaluate short-term clinical outcomes following SGB in patients with CRPS, migraine, neuropathic, and nociceptive pain. It also investigates the impact of demographic variables (age, gender, and body mass index) and procedural factors (imaging technique, medication choices, and complications) on response patterns. We hypothesized that migraine patients would show more favorable response patterns compared with other pain phenotypes. Given the limited number of studies directly comparing multiple pain types within the same cohort, this research aims to explore associations between pain phenotypes and observed response patterns.

## 2. Materials and Methods

### 2.1. Study Design and Participants

The research took place at the neurosurgery and algology departments of a tertiary referral hospital from 1 January 2024 through 1 March 2025. The research followed STROBE (Strengthening the Reporting of Observational Studies in Epidemiology) guidelines for observational studies to ensure methodological rigor and transparency in reporting. The Ethics Committee of Adana City Training and Research Hospital granted approval for the study through Meeting No. 13 and Decision No. 519, in accordance with the Helsinki and Good Clinical Practice (GCP) guidelines. Written informed consent was obtained from all participants, allowing the use of anonymized data for scientific research purposes.

A total of 109 patients were screened; 96 were included in the final analysis, and 13 were excluded due to not meeting the inclusion criteria or loss to follow-up. This study included patients aged 18 and older who experienced chronic pain lasting more than three months and failed medical treatment and had pain located in the head–neck area, upper extremity, or thoracic region with sympathetic pain syndrome. The SGB treatment decision process occurred at weekly multidisciplinary board meetings through combined assessments from algology and neurosurgery specialists. This study excluded patients who had psychiatric conditions, cognitive problems, active infections, or coagulation disorders; used anticoagulants; or showed hypersensitivity to local anesthetics or corticosteroids or failed to keep scheduled follow-up appointments. Only patients with complete 6-month follow-up were included in the final analysis; patients without complete follow-up were excluded.

### 2.2. Data Collection and Variables

This research obtained patient data, including age, gender, and body mass index (BMI). This study documented patient information regarding their pain types, attendance at follow-up appointments, and received treatments. The Visual Analog Scale (VAS) measured pain intensity from 0 to 10 cm, the Oswestry Disability Index (ODI) evaluated functional status from 0 to 100, and the SF-36 (36-Item Short Form Health Survey) questionnaire assessed health-related quality of life. This study monitored safety outcomes through both the rates of complications and their specific types. The research team performed assessments at four points in time: starting with baseline measurements, followed by evaluations at 1, 3, and 6 months. The research team monitored patients for 60 min after the procedure to identify immediate complications and checked for early adverse events at their one-week checkup. This study excluded participants who did not appear for their scheduled follow-up appointments. Responders were defined as patients achieving ≥30% (and ≥50%) reduction from baseline VAS at 6 months.

### 2.3. Procedural Details

**Figure 1:**
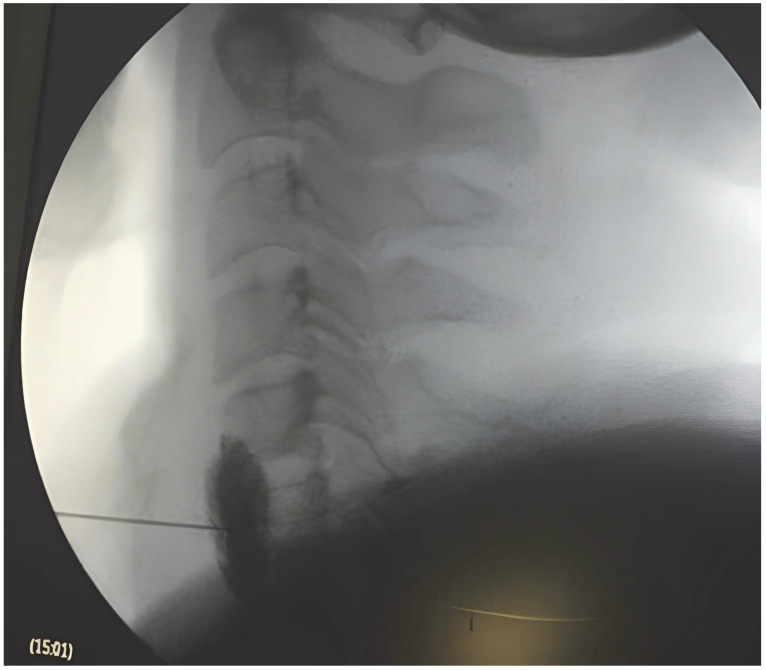
Lateral fluoroscopic image of stellate ganglion block (SGB), showing needle placement at the cervical level and contrast spread confirming accurate injection site.

The procedures took place in a specialized operating room that served as the dedicated space for interventional pain procedures under sterile operating conditions. The procedures required joint participation between an experienced interventional pain physician with more than 8 years of practice and a senior neurosurgeon who had performed surgery for over 25 years. The patients received treatment while lying on their backs with their neck slightly elevated. The C-arm system (Siemens Arcadis Avantic, Siemens Healthineers, Erlangen, Germany) provided fluoroscopic guidance for all block procedures. The procedure began with 1–2 mL of 2% lidocaine (Aritmal^®^, Osel Pharmaceuticals, Istanbul, Türkiye) local anesthetic before inserting a 22-gauge 5–8 cm needle into the C6 or C7 transverse process. The needle placement was verified through contrast injection (Figure 1) before injectate delivery at a controlled rate. During the study period, each patient received a single fluoroscopy-guided stellate ganglion block, and no additional sessions were performed. The treatment solution contained either 5 mL of 1% lidocaine (Aritmal^®^, Osel Pharmaceuticals, Istanbul, Türkiye)**,** 5 mL of 0.25% bupivacaine (Marcaine^®^, AstraZeneca, Södertälje, Sweden)**,** or 4 mL of local anesthetic with 1 mL of triamcinolone at 40 mg/mL concentration (Kenacort-A^®^, Bristol Myers Squibb, Princeton, NJ, USA)**.** The selection of injectate depended on patient characteristics, treatment background, and existing medical conditions [11,12]. The development of Horner’s syndrome symptoms, including ptosis, miosis, conjunctival hyperemia, and increased facial warmth, confirmed successful blockade within 5–10 min [13].

### 2.4. Post-Procedural Care and Follow-Up

The patients were monitored for sixty minutes following the procedure to identify any complications right away. The VAS measured pain levels, the ODI assessed functional status, and the SF-36 questionnaire evaluated quality of life. All outcomes were reassessed at the 1-, 3-, and 6-month follow-up visits. Early adverse events were additionally evaluated at the one-week visit. Patients who did not attend scheduled follow-up visits were excluded from the analysis.

### 2.5. Statistical Analysis

IBM SPSS Statistics version 26.0 (IBM Corp., Armonk, NY, USA) performed all statistical tests. The Shapiro–Wilk test evaluated the distribution patterns of continuous variables. This study presented normally distributed variables as mean values with standard deviations, while non-normal data were displayed as median values with interquartile ranges. The researchers presented categorical data as counts (%). The Mann–Whitney U test was used to compare two groups, while the Kruskal–Wallis test was applied for comparisons among multiple groups, with Bonferroni-adjusted post hoc tests performed when necessary.

The Friedman test was employed to evaluate longitudinal changes in VAS, ODI, and SF-36 scores within each group, and Wilcoxon signed-rank tests were conducted for pairwise comparisons between time points. The analysis of categorical data, including responder rates and complication frequencies, used the Chi-square test, or Fisher’s exact test when any expected frequency reached below 5. The analysis of VAS reduction in relation to ODI and SF-36 improvement used Spearman’s rank correlation coefficient. The Hosmer–Lemeshow goodness-of-fit test was used to check the calibration of the logistic regression model. Finally, this study used a two-tailed *p*-value of 0.05 or less to determine statistical significance.

## 3. Results

### 3.1. Demographic and Baseline Characteristics

This research included ninety-six participants in total and presents baseline demographic and clinical information about the patients in Table 1. The four pain subgroups showed no statistical differences in their age distribution, sex composition, or BMI values. There were no significant differences among subgroups for baseline variables (all *p* > 0.05).

### 3.2. Primary Outcome: Pain Intensity (VAS)

**Figure 2:**
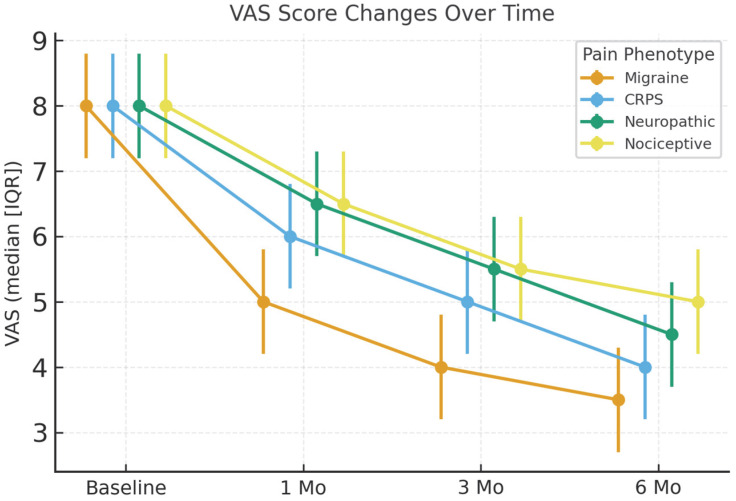
VAS score changes over time. VAS scores at baseline and at 1, 3, and 6 months across pain subgroups. Values are expressed as median (IQR). Between-group differences were analyzed using the Kruskal–Wallis test with Bonferroni-adjusted post hoc comparisons; within-group changes were analyzed with the Friedman and Wilcoxon signed-rank tests.

All treatment groups showed changes in VAS pain scores throughout the follow-up period. VAS scores decreased more in patients with migraine and CRPS compared with the neuropathic and nociceptive groups. At 6 months, median VAS reductions were 4.0 (3.5–5.0) for migraine, 3.7 (3.0–4.5) for CRPS, 3.4 (2.8–4.0) for neuropathic, and 3.0 (2.5–3.8) for nociceptive pain (all *p* < 0.001), as illustrated by the overall trend in Figure 2. The majority of migraine and CRPS patients reached significant pain relief according to responder rates, as shown in Table 1.

### 3.3. Secondary Outcomes: Functional Status and Quality of Life

**Figure 3:**
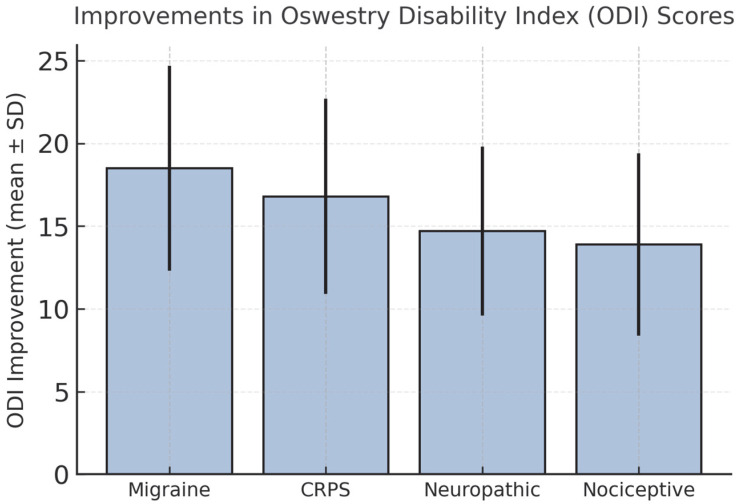
Improvements in Oswestry Disability Index (ODI) scores at 6 months across pain subgroups. Values are expressed as mean ± SD. Error bars indicate standard deviations. Differences among groups were analyzed using the Kruskal–Wallis test.

**Figure 4:**
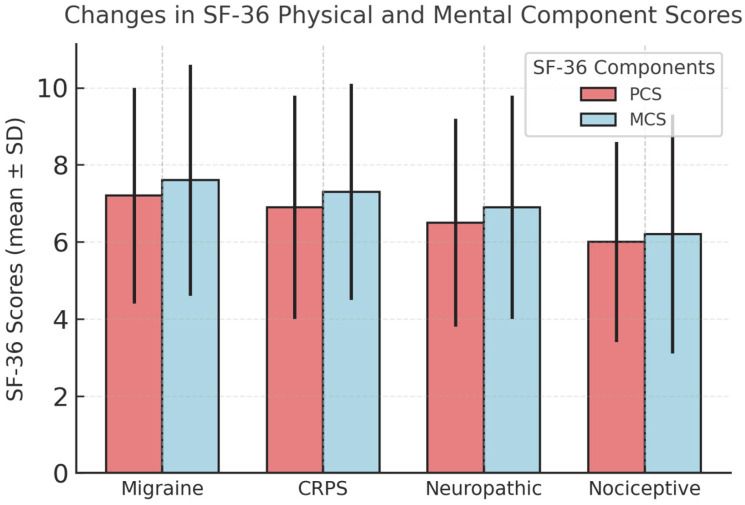
Changes in SF-36 Physical Component Score (PCS) and Mental Component Score (MCS) at 6 months across pain subgroups. Values are expressed as mean ± SD. Error bars indicate standard deviations. Differences among groups were analyzed using the Kruskal–Wallis test.

**Figure 5:**
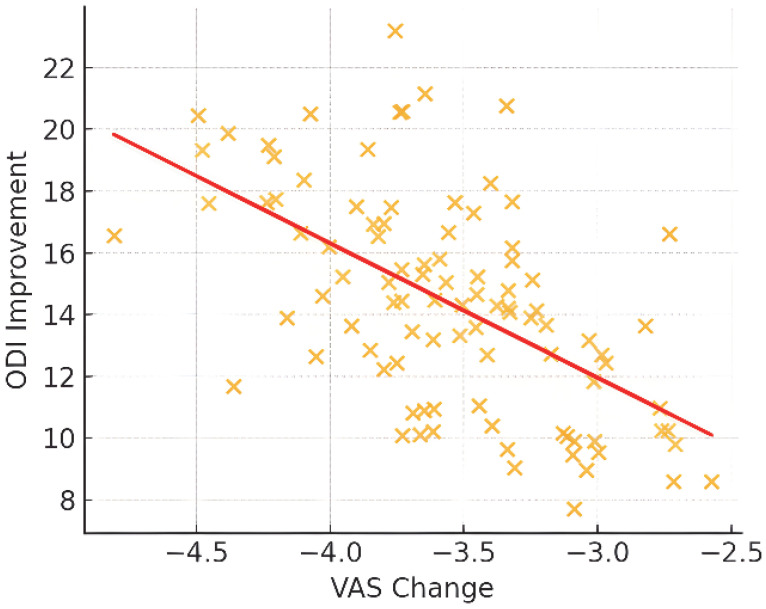
Scatter plot showing the correlation between VAS reduction and ODI improvement at 6 months, analyzed with Spearman’s rank correlation test.

The ODI scores showed the largest change in the migraine group, while SF-36 physical and mental component scores (MCS) increased in all groups without notable differences between subgroups (Table 2, Figure 3 and Figure 4). The analysis of different injectate regimens showed no apparent differences in observed outcomes, consistent with the study’s methodological approach. Correlations between VAS reduction and changes in ODI and SF-36 scores showed positive associations (Figure 5). Correlation analysis indicated a positive association between VAS reduction and changes in ODI and SF-36 scores, consistent with a moderate-strength relationship.

### 3.4. Multivariate Analysis of Treatment Response

We used a multivariate logistic regression analysis to find out which factors independently increased the chances of achieving clinically significant pain relief after a stellate ganglion block. The dependent variable was characterized as a ≥50% reduction in VAS score at the 6-month follow-up, while the independent variables comprised age, sex, BMI, pain phenotype, injectate type, and the occurrence of complications.

The overall model was statistically significant (χ^2^ = 19.6, *p* = 0.011) and elucidated 26.8% of the variance in responder status, as indicated by the Nagelkerke R^2^ coefficient. As shown in Table 3, pain phenotype was the only variable that was not related to treatment success. In comparison to patients with nociceptive pain, those suffering from migraine (OR = 2.85, 95% CI 1.21–6.71, *p* = 0.016) and CRPS (OR = 2.22, 95% CI 1.03–4.97, *p* = 0.041) exhibited a significantly higher probability of achieving a ≥50% reduction in VAS. Neuropathic pain exhibited a nonsignificant trend towards enhanced outcomes (OR = 1.65, 95% CI 0.82–3.28, *p* = 0.15). No demographic variables (age, sex, BMI), injectate type, or procedural complications demonstrated an independent association with responder status (all *p* > 0.05).

These results suggest that the observed response to stellate ganglion block may be influenced by the underlying pain phenotype rather than demographic or technical factors, supporting the potential role of sympathetic modulation as a key mechanism in migraine and CRPS.

### 3.5. Safety Outcomes

The safety evaluation showed that the procedure resulted in only temporary minimal complications (Table 4). The procedure was generally well tolerated, with no major adverse events observed.

During the 6-month observation period, stellate ganglion block was associated with sustained reductions in pain and improvements in function and quality of life, particularly among patients with migraine and CRPS. The procedure was generally well tolerated, with no major complications observed, and the choice of injectate did not appear to influence the observed outcomes.

## 4. Discussion

This research observed changes in outcomes associated with SGB in patients with various pain conditions, with the greatest changes noted among those with migraine and CRPS. The study results show that migraine patients exhibited changes in VAS scores exceeding the minimal clinically important difference (MCID), within the limitations of a retrospective design. The current study is consistent with earlier research reporting associations between ultrasound-guided SGB treatment and changes in headache frequency and disability ratings in patients with treatment-resistant migraines [14,15]. Hou et al. reported associations between ultrasound-guided SGB procedures and changes in migraine-related outcomes, which are consistent with the findings of the present study [14]. The existing evidence supports the use of SGB as a supplementary treatment for patients who do not respond to standard migraine medications [14,15].

The analgesic mechanism of SGB is multifactorial and primarily based on sympathetic modulation. SGB is associated with vasodilation, increased local blood flow, and reduced release of norepinephrine and inflammatory neuropeptides from sympathetic terminals due to temporary blockade of the cervical sympathetic chain [3,4,9]. This physiological alteration diminishes peripheral sensitization and stabilizes nociceptive transmission, resulting in a decreased perception of pain. Additionally, prior neuroimaging studies have shown that SGB affects central pain-processing regions, especially the hypothalamus and limbic networks, leading to central desensitization and enhanced autonomic balance [11,12]. These peripheral and central effects elucidate the rationale behind sympathetic block resulting in significant pain alleviation in conditions such as migraine and CRPS.

The VAS scores decreased by a median of –3.7 points (IQR 3.0–4.5) for CRPS patients, which matches previous research studies and meta-analyses that demonstrated significant pain relief through sympathetic modulation [2,9,16]. Recent meta-analytic evidence substantiated that CRPS patients undergo considerable pain alleviation and functional enhancement subsequent to SGB treatment [2]. A study of more than 200 patients demonstrated that SGB treatment led to better pain management and enhanced disability and functional abilities [16]. The research evidence supports SGB as a therapeutic option for CRPS treatment because it aligns with the established role of sympathetic blockade in this condition [2,9,16].

From a pathophysiological perspective, the disparate responses noted among pain phenotypes may be elucidated by the differing levels of sympathetic involvement. Migraine and Complex Regional Pain Syndrome (CRPS) exhibit elevated sympathetic activity, modified vasomotor reflexes, and heightened catecholamine sensitivity, rendering them more susceptible to sympathetic disruption [2,9,16]. On the other hand, somatic or inflammatory pathways mostly control neuropathic and nociceptive pain, while sympathetic modulation has a smaller effect. Consequently, the phenotype-specific associations observed in this study suggest inherent neurobiological differences and support the possibility that SGB may provide greater benefit in sympathetically maintained pain syndromes [9,10,11,12]. Recent systematic reviews and meta-analyses have further substantiated the analgesic and functional efficacy of ultrasound-guided stellate ganglion block across various pain conditions, corroborating the current findings [2,11,16].

Patients with neuropathic pain in our study showed changes in clinical outcomes, consistent with previous research on postherpetic neuralgia patients reporting improvements in sleep quality and life satisfaction [15,17,18]. Patients with nociceptive pain who received SGB experienced decreased pain levels, but the benefits were more restricted. This aligns with perioperative research showing that SGB decreases anesthetic and opioid requirements and modifies surgical physiological responses [4,19]. These more modest outcomes can be explained by the pathophysiological mechanisms of nociceptive pain, which are predominantly mediated by somatic and inflammatory pathways rather than by sympathetic overactivity. Since SGB primarily targets sympathetically maintained pain, its effect on purely nociceptive mechanisms is expected to be limited [4,19]. This observation underscores the importance of phenotype-specific differences in predicting treatment response. This study’s findings suggest that outcomes following SGB may differ according to pain type, with sympathetically mediated syndromes showing more favorable responses.

This study’s results show that different injectate combinations, which included lidocaine, bupivacaine, and local anesthetic–steroid mixtures, did not produce substantial variations in treatment outcomes [11,12]. The results from this study confirm the findings of Shah et al. and Vinyes et al., who established that SGB produces its therapeutic effects through sympathetic blockade regardless of the specific medications administered [11,12].

The procedure was generally well tolerated, with only minor transient side effects, such as hoarseness and conjunctival injection, all of which resolved without lasting effects. [13]. The study’s findings align with previous systematic reviews that reported few adverse effects in their results [13]. This study demonstrates that SGB treatment brought about pain relief, functional enhancement, and quality-of-life improvement through ODI and SF-36 score reductions [11,12]. SGB may be considered as an adjunctive intervention for further observational and prospective evaluation in patients with various pain conditions.

This research benefited from its substantial participant number, which enabled robust subgroup evaluations and the ability to study various pain patterns in one study population, which is uncommon in the published literature. It maintained methodological reliability through standardized procedures, multidisciplinary decision-making, and consistent follow-up assessments. However, several limitations must be recognized. As this study was conducted retrospectively at a single center, the results cannot be generalized to other settings. Participants may recall their past experiences differently, which could affect the data. The absence of a control or comparison group makes it difficult to evaluate the true treatment effect. The six-month research period was also insufficient to observe long-term effects, preventing us from determining the duration of treatment outcomes. This relatively short follow-up limits the assessment of long-term treatment durability and the possible recurrence of pain symptoms. Also, the fact that there was no blinding during the evaluation process may have led to bias in the observer or reporting. The results showed no significant impact of different injectate protocols, but further research should investigate standardized pharmacological treatment approaches [11,12]. In addition, more specific studies are required to examine different phenotypes, as this study demonstrates significant differences among them.

Cost-effectiveness is an important consideration for the clinical implementation of SGB. Although our study did not include an economic analysis, the rapid pain relief and functional improvements associated with SGB may reduce the need for prolonged pharmacological therapy and limit repeated healthcare utilization. These potential advantages highlight the importance of incorporating economic perspectives into future evaluations. A recent systematic review of pharmacological treatments for chronic migraine demonstrated that effective therapies can be cost-effective compared with conventional approaches [20]. Given the observed changes in the migraine subgroup, interventional strategies such as SGB may be considered for comprehensive economic evaluation.

Future research should include multicenter randomized controlled trials that evaluate SGB against sham procedures and standard treatments for various pain conditions. The assessment of treatment durability and repeated intervention effectiveness requires at least 12 months of follow-up. Patient selection for SGB can be improved through phenotype-stratified analyses that integrate demographic information, clinical data, and neurophysiological predictors. Comparative studies of pharmacological agents are needed to determine the most effective combination of injectate type, concentration, and volume. Finally, standardized complication reporting in multicenter safety registries will strengthen the risk–benefit evaluation of SGB and help define its role in clinical practice.

## 5. Conclusions

The research findings indicate that SGB was generally well tolerated and was associated with observed reductions in pain scores and functional limitations, particularly in migraine and CRPS. This research suggests that SGB may represent a viable interventional option for further prospective evaluation. Further prospective studies are warranted to confirm these observations and define the clinical role of SGB in different pain phenotypes.

## Figures and Tables

**Table 1:** Baseline characteristics and responder rates.

Variable	Migraine (*n* = 28)	CRPS (*n* = 30)	Neuropathic (*n* = 22)	Nociceptive (*n* = 16)
Age, mean ± SD	45.9 ± 11.8	46.8 ± 12.1	47.5 ± 11.2	48.1 ± 12.4
Sex (M/F)	13/15	16/14	11/11	10/6
BMI, mean ± SD	28.1 ± 3.4	27.7 ± 3.3	27.9 ± 3.2	28.0 ± 3.1
Responders ≥ 30%	85.7%	80.0%	72.7%	62.5%
Responders ≥ 50%	64.3%	56.7%	50.0%	37.5%

Note: SD = standard deviation; values are expressed as mean ± SD or *n* (%).

**Table 2:** Functional outcomes (ODI, SF-36) and injectate subgroup analysis.

Outcome	Migraine	CRPS	Neuropathic	Nociceptive
ODI improvement	+18.5 ± 6.2	+16.8 ± 5.9	+14.7 ± 5.1	+13.9 ± 5.5
SF-36 PCS Δ	+7.2 ± 2.8	+6.9 ± 2.9	+6.5 ± 2.7	+6.0 ± 2.6
SF-36 MCS Δ	+7.6 ± 3.0	+7.3 ± 2.8	+6.9 ± 2.9	+6.2 ± 3.1
Injectate subgroup analysis	No difference	No difference	No difference	No difference

Note: Values are expressed as mean ± SD. ODI = Oswestry Disability Index; SF-36 = Short Form-36. The Kruskal–Wallis test was used to compare pain subgroups, and no significant differences were observed between injectate subgroups (all *p* > 0.05).

**Table 3:** Multivariate logistic regression analysis identifying predictors of ≥50% reduction in VAS score at 6 months.

Variable	Odds Ratio (OR)	95% Confidence Interval	*p*-Value
Age	0.98	0.94–1.03	0.42
Sex (Female vs. Male)	1.12	0.58–2.17	0.74
BMI	0.97	0.88–1.07	0.53
Pain phenotype (Ref: Nociceptive)			
Migraine	2.85	1.21–6.71	0.016
CRPS	2.22	1.03–4.97	0.041
Neuropathic	1.65	0.82–3.28	0.15
Injectate type	1.09	0.61–1.93	0.76
Complication (Yes vs. No)	0.89	0.38–2.04	0.78

Note: OR = odds ratio; CI = confidence interval; CRPS = complex regional pain syndrome; BMI = body mass index; Ref = reference group. The Hosmer–Lemeshow test indicated an adequate model fit (*p* = 0.51), confirming that the logistic regression model appropriately represented the observed data.

**Table 4:** Safety outcomes.

Complication	*n* (%)
Transient hoarseness	6 (6.3%)
Conjunctival injection	3 (3.1%)
Major adverse events	0 (0%)

Note: Values expressed as *n* (%). This research includes descriptive statistical data. The Chi-square test revealed no meaningful relationship between complications and treatment outcomes.

## Data Availability

The data presented in this study are available on reasonable request from the corresponding author. The data are not publicly available due to privacy and ethical restrictions.

## Abbreviations

The following abbreviations are used in this manuscript:

Abbreviation	Full Form
SGB	Stellate Ganglion Block
CRPS	Complex Regional Pain Syndrome
VAS	Visual Analog Scale
ODI	Oswestry Disability Index
SF-36	36-Item Short Form Health Survey
BMI	Body Mass Index
STROBE	Strengthening the Reporting of Observational Studies in Epidemiology
GCP	Good Clinical Practice
PCS	Physical Component Score
MCS	Mental Component Score
MCID	Minimal Clinically Important Difference
